# Carbonation Resistance of Ternary Portland Cements Made with Silica Fume and Limestone

**DOI:** 10.3390/ma17112705

**Published:** 2024-06-03

**Authors:** Miguel Ángel Sanjuán, Esperanza Menéndez, Hairon Recino

**Affiliations:** 1 Spanish Institute of Cement and Its Applications (IECA), C/José Abascal, 53, 28003 Madrid, Spain; 2 The Eduardo Torroja Institute for Construction Science (Spanish National Research Council, CSIC), C/Serrano Galvache, 4, 28033 Madrid, Spain; emm@ietcc.csic.es (E.M.); h.recino@ietcc.csic.es (H.R.)

**Keywords:** sustainable materials, service life, carbonation, coarse silica fume, limestone

## Abstract

Ternary blended cements, made with silica fume and limestone, provide significant benefits such as improved compressive strength, chloride penetration resistance, sulfates attack, etc. Furthermore, they could be considered low-carbon cements, and they contribute to reducing the depletion of natural resources in reference to water usage, fossil fuel consumption, and mining. Limestone (10%, 15%, and 20%) with different fineness and coarse silica fume (3%, 5%, and 7%) was used to produce ternary cements. The average size of coarse silica fume used was 238 μm. For the first time, the carbonation resistance of ternary Portland cements made with silica fume and limestone has been assessed. The carbonation resistance was assessed by natural carbonation testing. The presence of coarse silica fume and limestone in the blended cement led to pore refinement of the cement-based materials by the filling effect and the C-S-H gel formation. Accordingly, the carbonation resistance of these new ternary cements was less poor than expected for blended cements.

## 1. Introduction

Cement manufacturing is a major contributor to anthropogenic global warming [1], which accounts for approximately 7.4% [2,3,4] of worldwide carbon dioxide emissions. Consequently, life cycle analyses have demonstrated that Portland cement is responsible for 60–80% of carbon dioxide emissions from concrete manufacturing [5] since the production of one ton of Portland cement clinker releases 800–1100 kg of carbon dioxide into the atmosphere for fuel combustion (white clinker: 480–560 kg CO_2_; gray clinker: 280–330 kg CO_2_) and for the calcination process (white clinker: 540–520 kg CO_2_; gray clinker: 530–520 kg CO_2_) [6]. Accordingly, we should be able to reduce carbon dioxide release associated with Portland cement production as far as possible with current know-how. Efforts to lower the carbon dioxide emissions from Portland cement manufacturing include alternative fuels and raw materials during Portland cement clinker production and low-carbon cements with a low clinker factor. In addition, for climate change mitigation purposes, a more specific and complete inventory may be needed by considering the carbon dioxide uptake by cement-based materials [7]. Through this, it would be possible to establish a more complete life cycle inventory of concrete in terms of carbon footprint [8,9,10,11,12]. Currently, some low-carbon concretes with embodied carbon dioxide below 100 kg/m^3^ CO_2 eq._ are available. This means a reduction of carbon dioxide by over 70% versus standard concrete made with CEM I.

Carbon dioxide uptake is the amount of mentioned gas that has been chemically bound by the cement paste constituents and pore solution contained in the hardened concrete. It should be expressed as the mass of bound carbon dioxide per square meter of the considered structure [13].

According to Pade and Guimaraes [14], concrete structures built in 2003 will be able to bind around 28% of the carbon dioxide emissions from cement production during the next 70 years. By contrast, Yang et al. [15] reported that concrete structures could bind, for a period of a hundred years, about 18–21% of the process carbon dioxide emissions. This percentage was lowered by Fitzpatrick et al. [16] to about 16% for a period of carbonation of one hundred years (concrete structures produced in 1972). The highest percentage of bound carbon dioxide reported in the literature was 43% [17] from a global study considering worldwide data from 1930 to 2013. A detailed comparison of some studies was reported elsewhere [6].

Carbonation of cement-based materials is a natural aging physicochemical process widely studied in the literature [11,18,19,20,21,22,23,24,25], in which carbon dioxide diffuses from the atmosphere through the mortar or concrete capillary pores and reacts with carbonatable products present in the pore solution or in the solid phase, such as calcium hydroxide known as portlandite, calcium silicate hydrate, named as C-S-H gel, ettringite, and to a lesser extent, anhydrous phases of clinker, specifically tricalcium silicate, C_3_S, and dicalcium silicate, C_2_S [24]. The main reaction is the reaction of calcium hydroxide, Ca(OH)_2_, with carbon dioxide, CO_2_, to form calcium carbonate, CaCO_3_ [10,18,19,26,27]. Then, the pH of the concrete pore solution decreases, and the risk of steel rebar corrosion increases [18,24,25,26]. Consequently, this process can negatively affect the durability of reinforced concrete on the one hand, but it can be a way to mitigate climate change impacts on the other [27,28,29,30]. In a nutshell, carbonation is a significant carbon dioxide sink that is not yet included in life cycle inventories.

Only a few studies were focused on the bound carbon dioxide quantification [18] and therefore, the estimation of carbon dioxide uptake remains a very challenging task. Andersson et al. proposed a simplified estimation method named Tier 1 [31], whereas the European standard EN 16757 defines an advanced method or Tier 2 in its Annex G, formerly known as Annex BB [13]. This European standard estimates the carbon dioxide uptake considering a direct relationship between carbonation and reactive CaO content in concrete. Several steps would have to be taken in order to estimate the carbon dioxide uptake, more specifically, (i) degree of carbonation, (ii) carbonation rate, which can be estimated from both the concrete compressive strength class and the field exposure conditions, and (iii) maximum theoretical carbon dioxide uptake in fully carbonated concrete estimated from the reactive CaO content in the binder.

Yang et al. [15] and Fitzpatrick et al. [16] found a good agreement between the results obtained by applying the experimental procedure and those calculated by applying the method defined in the European standard EN 16757. By contrast, Younsi et al. [32] found that the European standard’s method underestimates the carbon dioxide uptake of concretes made with additions since the carbonation depth was underestimated. In particular, the model underestimates both the carbonation rate of up to 61% [33] and the maximum theoretical carbon dioxide uptake of up to 77% for high content of ground granulated blast-furnace slag in the concrete. This fact was highlighted by Andrade et al. [30]. Furthermore, the degree of carbonation under indoor exposure was underestimated, while under outdoor exposure was overestimated [33]. Summing up, the method defined in the European standard EN 16757 works best under outdoor exposure [33]. Although full consensus is highly desirable, it would be particularly valuable to continue the comparison in natural carbonation, experimental versus estimated results. Currently, there is a lack of availability of experimental data to perform the comparison with theoretical models [18]. This is a very difficult challenge, indeed, particularly in the case of concrete made with blended cements.

Blended cements with a high content of Portland cement constituents, other than clinker, will contribute to the drawdown of carbon dioxide emissions and, therefore, the negative environmental impact of Portland cement production. In an attempt to produce low-carbon and cost-effective blended cements, new formulations are currently being investigated [34]. Zeraoui et al. [35] studied ternary binders (Portland cement-ground granulated blast-furnace slag-flash-calcined sediment) and reported that 10% flash-calcined sediment plus 40% ground granulated blast-furnace slag can replace 50% of Portland cement. However, the water demand increases with the use of flash-calcined sediment, but it enhances the compactness and density of the mortar.

The rheological properties of ternary cements made of coal fly ash, silica fume or limestone powder and Portland cement were assessed by Srinivas et al. [36]. They found that silica fume and limestone, ranging from 5% to 10%, improved the buildability. Zhao et al. [37] reported the enhancement of the packing density of ground slag-silica fume-cement pastes and their increase of 28-days compressive strength.

Silica fume, fly ash, and limestone powder were used to produce Ultra-high-performance concrete by Li et al. [38] to model their pozzolanic reactions. Silica fume showed the strongest effect on the compressive strength, followed by the coal fly ash and limestone powder. In addition, nanosilica (2%), calcined clay (23%), and Portland cement (75%) promote the conversion of macropores into mesopores [39]. However, Papatzani and Paine [40] found that 10% of silica fume addition to ternary systems Portland cement-limestone-fly ash could be excessive since unreacted particles were observed by SEM. Ultra-high-performance fiber-reinforced concrete (UHPFC) can be made by using ternary cement with silica fume and limestone, as reported by Kang et al. [41]. According to Li et al. [42], the limestone promotes a plasticization effect; furthermore, they reported that the optimal content of limestone powder for UHPFC is 50% by volume.

**Figure 1:**
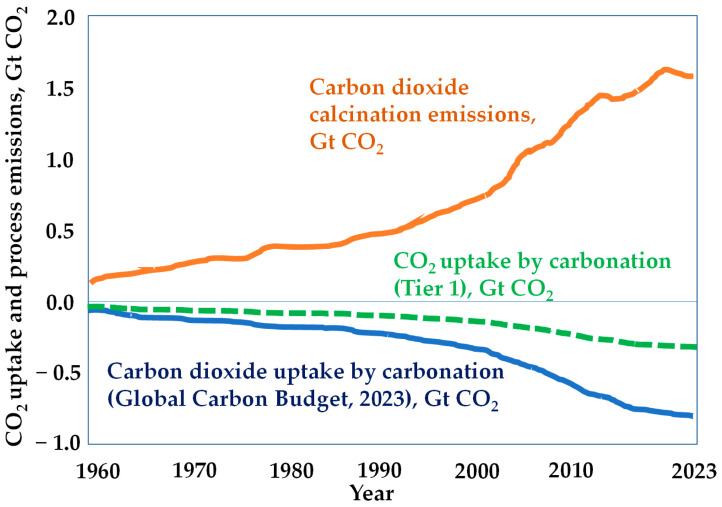
Global carbon dioxide uptake by cement-based materials from 1960 to 2023, reported by the *Global Carbon Budget* report, and the carbon dioxide uptake estimated by the Tier 1 methodology «source: Ref. [7]».

Natural carbonation, also known as (re-)carbonation, permanently stores carbon dioxide. One point that should be underlined, particularly within the climate change context, is that the carbonation rate of blended cements is faster than that of CEM I cements. Therefore, this fact should be clearly considered in *The Seventh Assessment Report of the Intergovernmental Panel on Climate Change* (IPCC). Figure 1 shows the first attempt made by the *Global Carbon Budget* report [43] to estimate the (re-)carbonation from 1960 to 2023, i.e., carbon dioxide uptake by cement-based materials (line blue in Figure 1). It means that above 700 Mtons/year could be attributed to the carbonation sink. This estimation is compared to the carbon dioxide uptake estimated by the Tier 1 methodology (dashed line green in Figure 1) defined and calculated in reference [7].

Accordingly, a ternary cement made with Portland cement clinker, limestone powder, and a third cement constituent, for example, coarse silica fume, can be a promising construction product due to the good experience [34,44], the adequate availability of the constituents, and the low environmental impact.

Ground limestone is already being used worldwide in Portland cement, providing some characteristics to the cement depending on its particle size distribution (PSD) and its content in the blended cement, i.e., dilution, filler, nucleation, and chemical effects [34].

Silica fume is the world’s most widely used ultrafine particles for cement and concrete production because of their good durability and mechanical properties. This is explained by the optimization of the packing density [45,46] and, above all, by the pozzolanic reaction, i.e., portlandite is consumed to produce additional C-S-H gel with a lower Ca/Si ratio [44,47], and providing nucleation sites to C-S-H gel.

In view of these considerations, the carbonation resistance of clinker–limestone–silica fume ternary cements is assessed. Furthermore, this paper provides data on carbon dioxide uptake due to the natural carbonation of clinker–limestone–silica fume ternary cements. This kind of information is currently lacking in the scientific literature, particularly for concretes made with blended cements. These data are required to evaluate the actual environmental impact of concrete more accurately in civil engineering and building.

In the following, the natural carbonation results of this experimental research program are discussed to assess the effect of both ternary cement constituents’ nature and curing conditions on carbonation resistance, carbon dioxide uptake of ternary cement mortars and service life estimation. For the first time, the carbonation resistance of ternary Portland cements made with silica fume and limestone has been assessed.

## 2. Materials and Methods

### 2.1. Materials

The experimental study was carried out on concretes designed with CEM I as per the European standard EN 197–1 [48] from Holcim España, Villaluenga de la Sagra, Toledo, Spain, and blended cements containing silica fume or limestone manufactured by mixing silica fume (H) from Ferroglobe PLC, Sada, Spain, and limestone (L) supplied by Holcim España, Villaluenga de la Sagra, Toledo, Spain with the Portland cement (C). The limestone was ground to reach three different fineness (given as percentage retained on the 120 µm sieve): 10% (8001 cm^2^/g), 20% (25,857 cm^2^/g), and 50% (25,954 cm^2^/g). Grinding times were 10, 20, and 50 min, respectively. Table 1 shows the chemical analysis of the samples. Most of the elements were analyzed using the molten pearl X-ray fluorescence technique, with a wavelength scattering X-ray spectrometer, Bruker’s S8 Tiger.

Loss on ignition (LOI) and sulfate content determination are described in the European standard EN 196-2 [49]. The alkali content (Na^+^ and K^+^) in cement, limestone, and coarse silica fume was determined by inductively coupled plasma optical emission spectrometry (ICP-OES) with a Varian model 725-ES equipment.

In addition, a siliceous sand (0/4 mm) from IETcc, as per the European standard EN 196-1 [50], was used to manufacture the mortars.

The ternary mix design is given in Table 2, and the criterion of coding is as follows:

- Reference: CEM I 42.5 R (100wt% CEM I).
- H: Silica fume content (0wt%, 3wt%, 5wt%, 7wt%).

LX1-X2-X3: Limestone content corresponding to a sieve non-passing fraction at a mesh width of 120 µm of not more than 10% by weight (X1), 20% (X2), and 50% (X3).

Since the silica fume has a high SiO_2_ content (96%), most standards limit its content in cement to less than 10%. Accordingly, the coarse silica fume content has been distributed between 0% and 10% (3%, 5%, and 7%). Regarding the limestone, it was pretended to simulate CEM II/A-L and CEM II/A-M and, therefore, the following amounts have been chosen: 15%, 20%, and 30%.

These new cements were used to manufacture prismatic mortar specimens (40 mm × 40 mm × 160 mm) with a cement-to-sand ratio of 1:3 and a water-to-cement ratio of 0.5 with distilled water and CEN standard sand [49]. Mortar mixing, molding, and curing are defined in the European standard EN 196-1 [49].

Mortar specimens were cured under lime water for 0, 3, or 28 days, rich in alkaline ions. The reason for adding lime to the curing water is to prevent the leaching out of the mortar pore solution. Limewater is a saturated aqueous solution of calcium hydroxide, which is sparsely soluble at room temperature in water (1.5 g/L at 25 °C).

### 2.2. Natural Carbonation Testing

**Figure 2:**
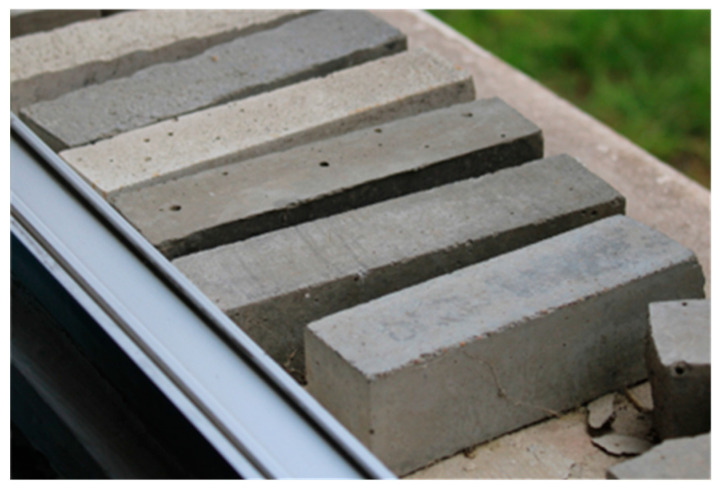
Carbonation depth testing made by exposing specimens in natural atmosphere.

The mortars were subjected to 2 years of natural carbonation (Figure 2) under sheltered conditions in the lab (40–50% RH and 25 °C), following the procedure defined in reference [24]. During the natural carbonation exposure period, the progress of carbonation was monitored by regular carbonation depth measurements of 28, 90, 180, 270, 365, and 730 days.

The mortar specimens were cut into strips of 20 mm size. Cutting with a saw in a horizontal position is suitable for determining the carbonation depth in a reliable, flexible and inexpensive manner [23]. The depth of carbonation was measured on the freshly sawn surface, which was previously cleared of dust and loose particles by spraying a mist of the phenolphthalein indicator solution (1% by weight) [51,52], which is colorless below pH 8.5 and attains a purple hue above pH 9.0.

## 3. Results and Discussion

### 3.1. Carbonation Depth

Following the procedures given by RILEM [51] and provided in the CEN/TS 12390-10 Technical Specification [52], it is proposed to assess several aspects related to the carbonation resistance of these ternary cements, such as the carbonation depth, carbonation rate, and CO_2_ uptake.

**Figure 3:**
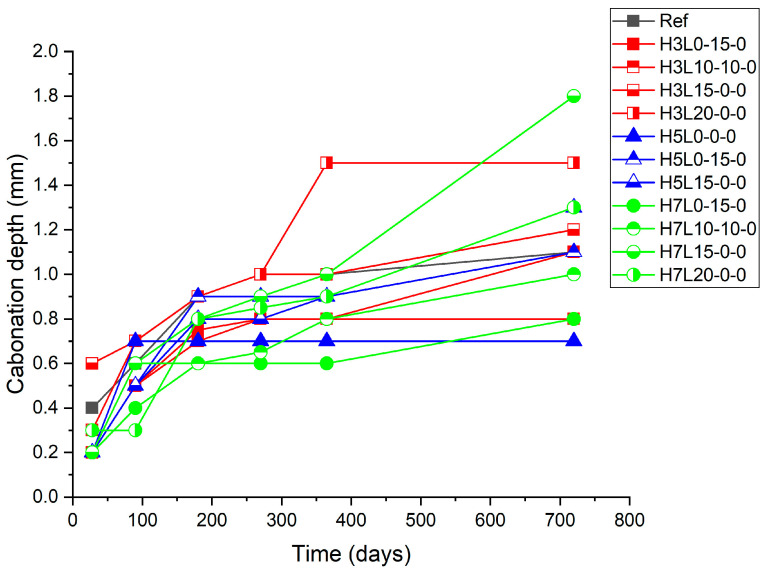
Carbonation depth of uncured samples.

**Figure 4:**
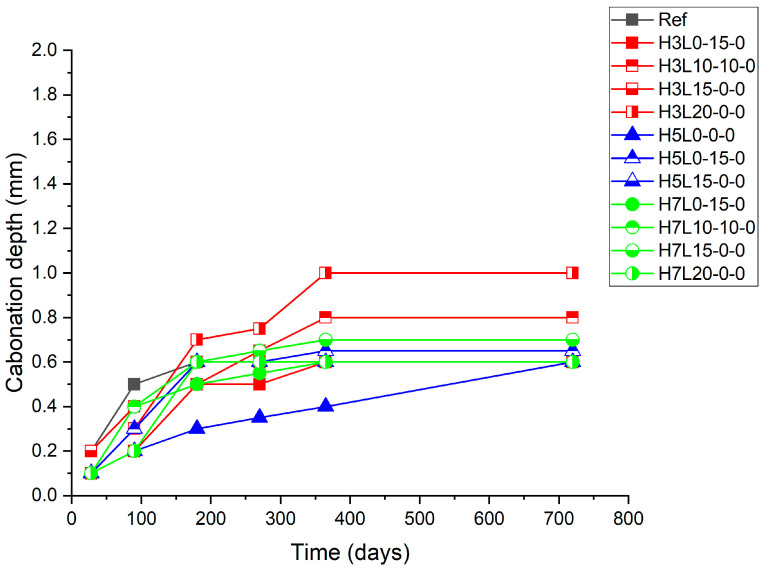
Carbonation depth of samples cured for 3 days.

The mortars were subjected to two years of natural carbonation under sheltered conditions. During the natural carbonation exposure period, the progress of the mortar carbonation was monitored by regular carbonation depth measurements of 28, 90, 180, 270, 365, and 730 days (Figure 2, Figure 3 and Figure 4).

**Figure 5:**
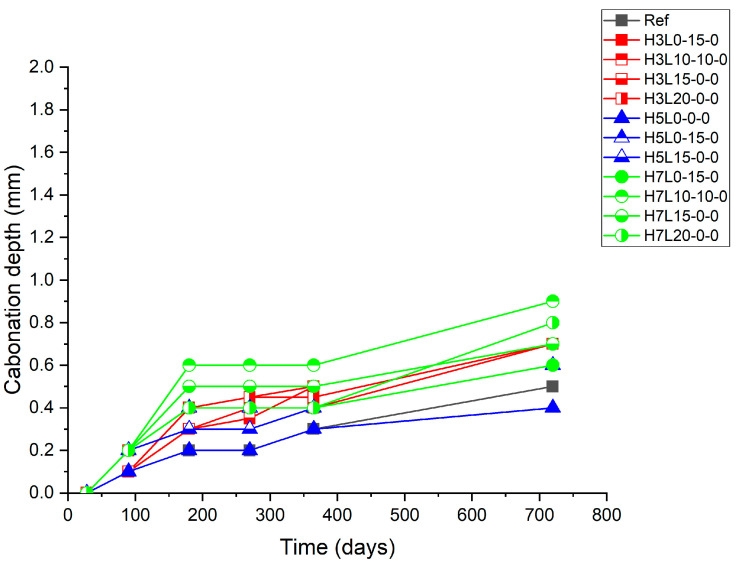
Carbonation depth of samples cured for 28 days.

Figure 3 shows the carbonation depth increase with the time of uncured samples, while Figure 4 and Figure 5 show the carbonation depth increase with the time of cured samples for 3 days and 28 days, respectively. As expected, with regard to the effect of the curing time on the carbonation resistance, the longer, the better [23,24].

For samples without curing, the lowest carbonation depth was found in specimens made with the highest silica fume content (5% and 7%), i.e., H5L0-0-0 and H7L0-0-0, followed by the reference cement made without any addition. This finding confirms the positive effect of the pozzolanic reaction of the silicon oxide present in the silica fume and the calcium oxide formed in the cement hydration [39]. This reaction leads to a denser structure [34]. However, the worst performance was found in the sample with the highest content of limestone (20%), H7L20-0-0 and H5L20-0-0, independently of the silica fume content (7% and 5%, respectively). This fact shows that bad curing affects limestone cements more negatively than silica fume ones. Accordingly, the use of silica fume in ternary cements made with limestone cannot improve the performance of limestone cements regarding the carbonation resistance of ternary cements.

Figure 4 shows that a three-day curing significantly enhances the carbonation resistance. In this case, cement with the lowest content of silica fume (3%) and the highest amount of limestone (20%), H3L10-0-0 and H3L20-0-0, presents the largest carbonation depth. This means that the increase of the silica fume content from 3% to 5% or 7% improves the performance (carbonation resistance).

Finally, samples cured for 28 days exhibited carbonation depths between 0.4 mm and 1 mm after 730 days of natural carbonation, as well as the samples cured for 3 days.

Test results for ternary cement carbonation have shown that curing conditions, particularly curing time, have a significant effect on the performance of samples. Accordingly, curing conditions should be optimized in relation to their performance [24]. Regarding the present results, the absence of wet-curing affects in a different way to the tested samples; indeed, at 28 days of curing for non-wet-curing, carbonated depths vary several tenths of a millimeter. Increasing the curing period to three days is sufficient for mortars with a high silica fume content; for others, curing must be longer.

Furthermore, the curing effect also depends on the ternary cement mix design. For example, increasing the curing period improves the carbonation resistance of mortars made with limestone. Accordingly, three days of curing could be enough for ternary cements made with silica fume and limestone.

**Figure 6:**
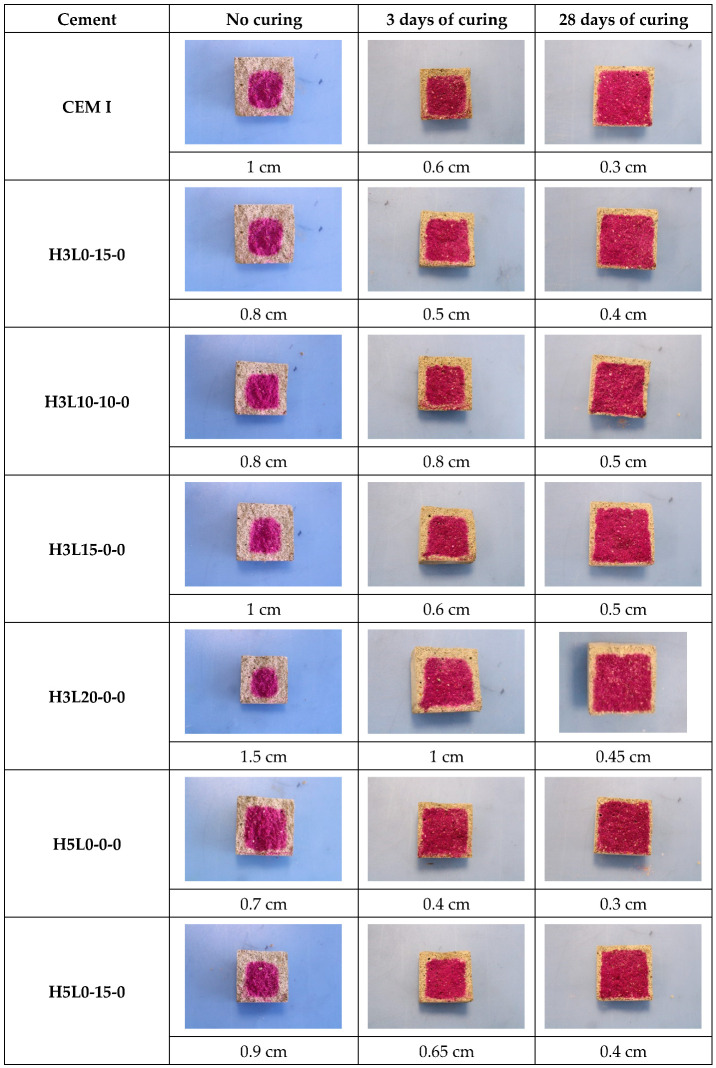

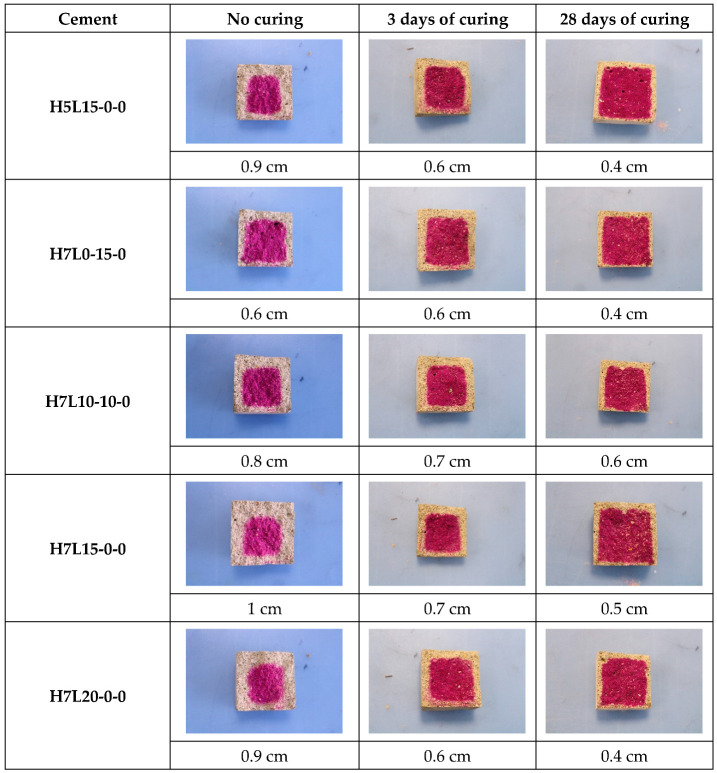
Carbonation depth of samples after 365 days of natural carbonation.

Figure 6 shows the carbonation depth measured on samples exposed to 365 days of natural carbonation. The phenolphthalein indicator solution was applied to the fresh fracture surface of the mortar. When the pH is above 8.6, the phenolphthalein indicator solution turns purple. By contrast, the pH of the mortar is below 8.6, where the phenolphthalein indicator remains colorless, suggesting the carbonation of the mortar.

The pH of the CEM I mortar pore solution is normally about 13–14 since it is saturated with calcium hydroxide and also consists of sodium and potassium hydroxide. Ternary cements are less alkaline than sound mortar (CEM I); therefore, they should have lower pH values of around 10–12 [16,18]. However, it is clearly a strong color change with the phenolphthalein indicator, as shown in Figure 6. Nevertheless, the phenolphthalein indicator solution procedure is frequently said to underestimate the actual carbonation depth since the color change occurs only when the pH drops below 9.8 for phenolphthalein [53]. In addition, Schultheiß et al. [54] found that some models, such as crack influence factor (CIF) approaches, underestimate the carbonation depth with increasing exposure time.

According to Zhang et al. [55], confocal Raman microscopy (CRM)-CaCO_3_ maps for measuring carbonation depth in cement-based materials present the same carbonation depth results to phenolphthalein solution, i.e., the average difference between both methods, about 1.2% [55]. Nevertheless, phenolphthalein is less reliable for low carbonation depths since it underestimates the true value. This fact mainly happens in short-term natural carbonation tests. Furthermore, Zhang et al. [55] suggested that phenolphthalein color change correlates well with the depletion of portlandite measured with confocal Raman microscopy (CRM) and other techniques. By contrast, Shi et al. [56] reported that the carbonation depth measured with phenolphthalein reflects the depletion of C-S-H gel with high calcium indicated by using thermodynamic modeling.

The carbonation fronts shown in Figure 5 are sharp and reflect a gradual transition. By contrast, some authors [57,58] found non-pronounced carbonation fronts. It should be highlighted that non-cured mortars exhibited the highest level of dispersion in the carbonation depth. On the other hand, in both the 3-day curing condition and the 28-day curing condition, the carbonation rate increases with the higher silica fume content in the mortars. This fact can be attributed to the decrease in portlandite since it reacts with the reactive silicon present in the silica fume [9,21], which is an amorphous, highly reactive pozzolan, to form a new C-S-H gel with low Ca/Si ratio [21]. Therefore, the pH of the pore solution decreases. Furthermore, silica fume helps in accelerating the hydration of C_3_S, C_2_S, and C_4_AF [34].

### 3.2. Carbonation Coefficient

Some models can be found in the literature for depicting the carbonation of cement-based materials [59], and most of them proceed by diffusion. Accordingly, the carbonation rate can be estimated from the carbonation depth measurements. Therefore, carbonation results have been modeled and analyzed according to Equation (1), where A (mm/s^0.5^) is the carbonation coefficient [21,60]. This parameter is assumed to be constant [4,5,8]. Nevertheless, it depends on the environmental relative humidity, pore size distribution (PSD), hydration degree, carbon dioxide concentration, and binder composition, among others [61].

X = A √t(1)

where X is the carbonation depth (mm), and t is the natural carbonation exposure time (year).

**Figure 7:**
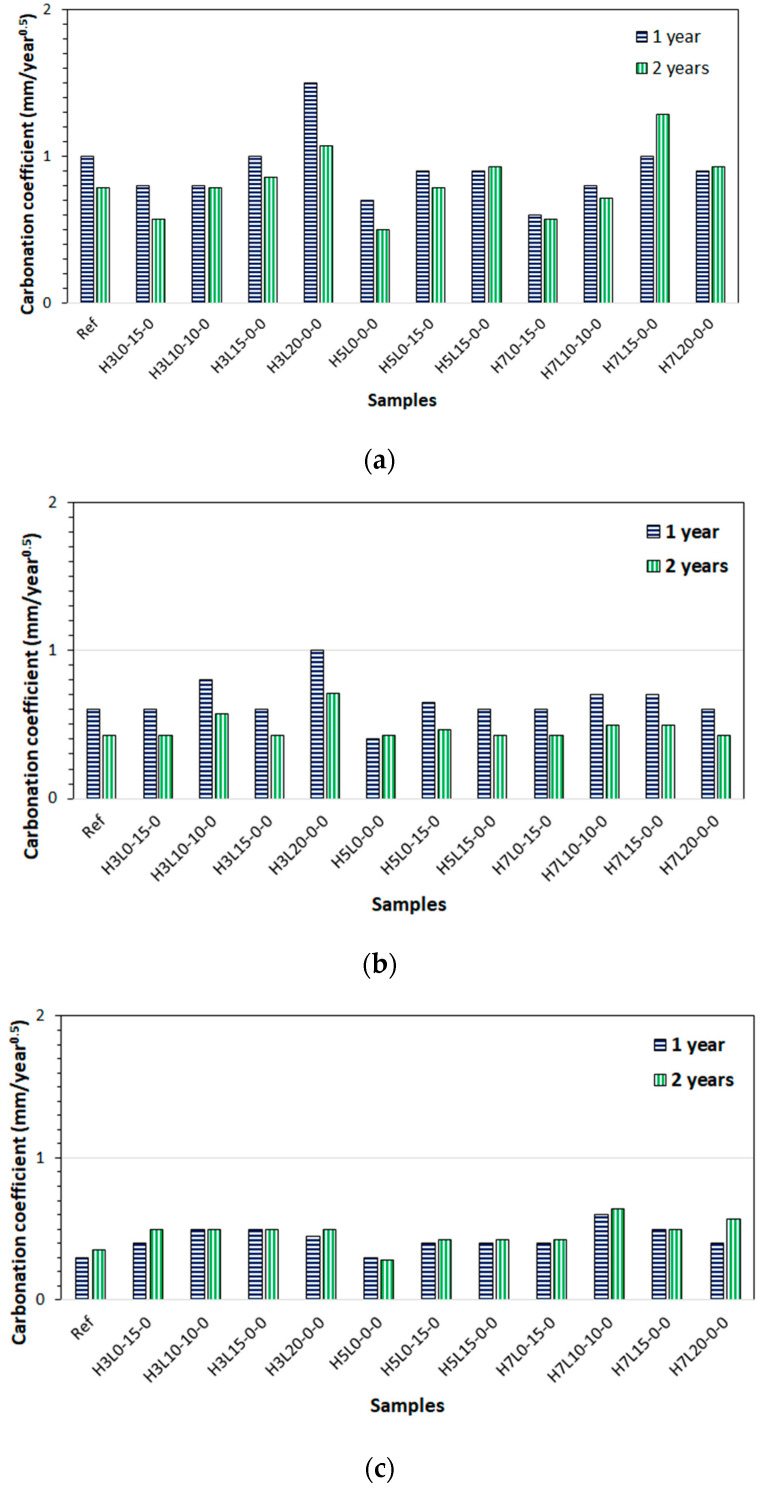
Carbonation coefficient calculated from the carbonation depth results measured at 365 and 730 days of natural carbonation of mortars: (**a**) without wet curing; (**b**) cured under water for 3 days; (**c**) cured under water for 28 days.

Figure 6 shows the carbonation coefficient, A, calculated from the carbonation depth results measured in mortars without wet curing (Figure 7a) or cured for 3 days (Figure 7b) or 28 days (Figure 7c), at 365 days and 730 days of natural carbonation. As expected, the longer the curing period, the better the carbonation resistance, i.e., lower carbonation coefficient [22,23,24].

In most cases, the carbonation coefficient, A, calculated from the carbonation depth results measured in mortars after 365 days of exposure, was higher than the one calculated with the results taken after 730 days of exposure. This finding was especially pronounced in the mortars without curing or those that were cured for 3 days, while in the mortars cured for 28 days, we did not observe significant differences. This finding suggests that it would be necessary to constantly assess the carbonation coefficient if the cement-based material was not properly cured. As expected, there is a direct relationship between the carbonation depth and the carbonation coefficient at a certain age given by Equation (1).

### 3.3. Service Life Estimation

The Eurocodes are a series of 10 European standards coded from EN 1990 to EN 1999 [62], providing a common European approach for the design of civil engineering works, buildings and other construction products. Eurocode 2 (EN 1992) “Design of concrete structures” [62] specifies technical rules for the design of concrete, reinforced concrete, and prestressed concrete structures, using the limit state design philosophy. In particular, EN 1992-1-1 [62] deals with the rules and concepts for serviceability, safety, and durability of reinforced concrete structures [62]. This European standard considers the durability and cover to reinforcement requirements with regard to the carbon dioxide ingress rate and concrete cover. Chapter 6.4 defines the “exposure resistance classes, ERC” and classifies concrete with respect to resistance against corrosion induced by carbonation (class XRC). In addition, Annex P provides an alternative cover approach for durability without the use of ERC as defined in Chapter 6.4; in this case, the deemed-to-satisfy approach given in EN 206 is followed [63]. Table 3 shows the minimum concrete cover depth for carbon reinforcing steel required for corrosion induced by carbonation according to Eurocode 2, Annex P [64]. The recommended Structural Class is S4 for the standardized compressive concrete strengths (XC1: C20/25; XC2: C25/30; XC3, and XC4: C30/37) and design working life of 50 years.

Natural carbonation results on ternary Portland cement mortars made with silica fume and limestone will be used to assess the potential for improvement of this new type of cement in comparison with the reference cement (CEM I). Furthermore, the minimum concrete cover required to prevent corrosion against carbonation specified by Eurocode 2, shown in Table 3, is taken as a benchmark figure. Since natural carbonation in the present study was performed at 60%RH and sheltered from rain, the corrosion exposure class induced by carbonation corresponds to the one coded as XC3 (Table 3). Accordingly, an S1 structural class requires a minimum cover thickness of 10 mm.

Table 4 shows that the carbonation depth estimated for 100 years of service life is lower than 10 mm for all the mixes when the mortars are cured for at least three days. By contrast, H3L20-0-0 and H7L15-0-0 mortars without curing exhibited carbonation depths of 10.7 mm and 12.8 mm, respectively. Therefore, these ternary cements comply with the requirements set out in the specification for S2—S6 structural classes (corrosion exposure class: XC3).

The choice of an adequate mix design for carbonation-induced reinforcement corrosion protection requires the consideration of several factors: composition (cement type), curing conditions, and exposure class (XC). In addition, the European standard EN 206 [63] specifies three strength classes: C20/25 for XC1, C25/30 for XC2, and C30/37 for XC3 and XC4. Consequently, the durability design of reinforced concrete structures commonly utilizes the deemed-to-satisfy rules concept, i.e., concrete mix design and concrete cover, which is mainly based on experience. This approach works well for traditional materials for which longtime experience is at the disposal of engineers. However, new Portland cement constituents and mix designs need assessment based on performance testing.

Nowadays, the exposure resistance classes (ERC) concept is proposed to classify concrete with regard to the resistance against corrosion induced by carbonation (XRC class). This system follows a performance-based concrete approach. Considering the exposure resistance classes given in EN 206 [63] and shown in Table 3 for corrosion induced by carbonation, the quality of the concrete given by the maximum carbonation coefficient (mm/year^0.5^) and concrete cover (mm) are set up in the structural project.

Table 5 provides the maximum carbonation coefficient and concrete cover required for the exposure resistance classes (ERC) given for the XC3 exposure class and a design service life of 50 years defined in Chapter 6.4 of the EN 1992-1-1 [62].

All the mortar mixes cured for 28 days, with carbonation coefficients below 0.6 mm/year^0.5^, can be used for all the exposure resistance classes (ERC). A similar conclusion can be reached for the mortars cured for three days, considering the carbonation coefficients obtained after two years of exposure. However, the carbonation coefficients calculated after one year of exposure are higher than the previous ones. Therefore, XRC 0.5 must be excluded. In addition, all the mortars without curing have carbonation coefficients over 0.6 mm/year^0.5^, but only two over 1.2 mm/year^0.5^, H3L20-0-0 and H7L15-0-0. Therefore, XRC 0.5 is excluded for all the cases, and XRC 1 is only excluded in two cases (Table 5).

Finally, the carbonation of cement-based materials is considered by the cement sector as a lever to reach carbon neutrality by 2050 [1,2,6,8,16]. In addition, carbon dioxide uptake by mortars and concretes has recently been included in the *Global Carbon Budget* report [43] since natural carbonation stores permanently carbon dioxide. Furthermore, these blended cements usually absorb more carbon dioxide than CEM I [7,9,11,18]. Then, appropriate methodologies for future concrete mix design should consider durable and sustainable aspects, i.e., to ensure the reinforced concrete service life but also to account for carbon dioxide uptake [6].

## 4. Conclusions

In order to minimize climate change, the cement sector needs to develop new cements with a high addition content, such as ternary cements. For the first time, the carbonation resistance of new ternary cements made with silica fume and limestone was assessed by means of natural carbonation testing.

The results presented in this paper confirm that blended cements have a higher carbonation rate than CEM I. This finding suggests that appropriate methodologies for future concrete mix design should consider durable and sustainable aspects, i.e., to ensure the reinforced concrete service life, but also to account for carbon dioxide uptake within the climate change context.

Furthermore, the longer the curing period, the better the carbonation resistance. This finding is in agreement with the results reported in previous literature regarding other types of cements. It is well known that limestone and silica fume in blended cements led, separately, to pore refinement of the cement-based materials by the filling effect and the C-S-H gel formation, respectively. Both effects can justify that the carbonation resistance of these new ternary cements was less poor than expected.

In particular, it should be highlighted that the carbonation coefficient, A, calculated from the carbonation depth results measured in mortars after 365 days of exposure, was higher than the one calculated with the results obtained after 730 days in the mortars without curing or those that were cured for three days. In the mortar mixes cured for 28 days, there were hardly any differences found. Then, it is concluded that for studies conducted in bad curing conditions, the carbonation coefficient, A, should be considered from results at longer ages.

Finally, it should be pointed out that all the mortar mixes cured for 28 days present carbonation coefficients below 0.6 mm/year^0.5^. Therefore, they can be used for all the exposure resistance classes (ERC) given in the European standard EN 206.

## Figures and Tables

**Table 1:** Chemical composition of silica fume (H), limestone (L), and CEM I (%) [34].

Chemical Composition (%)	CEM I	H	L	Physical Properties of CEM I	
SiO_2_	20.0	96.1	3.4	Specific gravity (g/cm^3^)	3.11
Al_2_O_3_	4.5	0.2	1.6	Initial setting time (min)	160
Fe_2_O_3_	2.7	0.1	0.4	Final setting time (min)	240
CaO	63.0	0.4	46.3	Volume expansion (mm)	0.0
MgO	1.9	0.1	0.3	Specific surface Blaine (cm^2^/g)	3811
SO_3_	3.1	0.1	0.1		
K_2_O	0.9	0.4	0.2	**Compressive Strength (MPa)**	
Ti_2_O_5_	0.2	0.0	0.1	1 days	14.32
P_2_O_5_	0.1	0.0	0.0	7 days	50.50
LOI	3.2	2.4	47.5	14 days	55.28
Na_2_O	0.3	0.2	0.1	28 days	59.25
CI^−^	0.1	0.0	0.0		

**Table 2:** Ternary cement mix design: silica fume (H), limestone (L) and cement (CEM I 42.5 R).

Cement Mix Code	CEM I (%)	H (%)	Total of Limestone (%)	10% Retained Limestone (%) (8001 cm^2^/g)	20% Retained Limestone (%) (25,857 cm^2^/g)	50% Retained Limestone (%) (25,954 cm^2^/g)
Reference	100	0	0	0	0	0
H3L15-0-0	82	3	15	15	0	0
H3L20-0-0	77		20	20	0	0
H3L0-15-0	82		15	0	15	0
H3L10-10-10	67		30	10	10	10
H5L0-0-0	95	5	0	0	0	0
H5L0-15-0	80		15	0	15	0
H5L15-0-0	80		15	15	0	0
H7L0-15-0	78	7	15	0	15	0
H7L15-0-0	78		15	15	0	0
H7L20-0-0	73		20	20	0	0
H7L10-10-0	68		25	10	10	5

**Table 3:** Minimum concrete cover depth (mm) for corrosion induced by carbonation for carbon reinforcing steel [64].

Environmental Requirement for Minimum Concrete Cover Depth (mm)	Structural Class					
Exposure Class: Corrosion Induced by Carbonation	S1	S2	S3	S4	S5	S6
XC1—Dry or permanently wet	10	10	10	15	20	25
XC2—Wet, rarely dry	10	15	20	25	30	35
XC3—Moderate humidity	10	15	20	25	30	35
XC4—Cyclic wet and dry	15	20	25	30	35	40

**Table 4:** Estimation of the carbonation depth (mm) for 100 years’ service life (Exposure Class: Corrosion induced by carbonation).

	Curing Time/Carbonation Depth (mm)		
Mortar Code	0	3	28
Reference	7.8	4.3	3.6
H3L0-15-0	5.7	4.3	5.0
H3L10-10-0	7.8	5.7	5.0
H3L15-0-0	8.5	4.3	5.0
H3L20-0-0	10.7	7.1	5.0
H5L0-0-0	5.0	4.3	2.8
H5L0-15-0	7.8	4.6	4.3
H5L15-0-0	9.3	4.3	4.3
H7L0-15-0	5.7	4.3	4.3
H7L10-10-0	7.1	5.0	6.4
H7L15-0-0	12.8	5.0	5.0
H7L20-0-0	9.3	4.3	5.7

**Table 5:** Maximum carbonation coefficient (mm/year^0.5^) and minimum concrete cover (mm) for carbon reinforcing steel required for the exposure resistance classes (ERC) given for the XC3 exposure class and a design service life of 50 years [64].

Exposure Resistance Classes (ERC)	XRC 0.5	XRC 1	XRC 2	XRC 3	XRC 4	XRC 5	XRC 6	XRC 7
Cover (mm)	10	10	15	20	25	25	35	40
Maximum carbonation coefficient (mm/y^0.5^)	0.6	1.2	2.4	2.7	3.6	4.5	5.4	6.4

## Data Availability

Data are contained within the article.
